# An Advanced, Silicon-Based Substrate for Sensitive Nucleic Acids Detection

**DOI:** 10.3390/s18093138

**Published:** 2018-09-17

**Authors:** Salvatore Petralia, Nunzio Vicario, Giovanna Calabrese, Rosalba Parenti, Sabrina Conoci

**Affiliations:** 1 STMicroelectronics Stradale Primosole, 50, 95121 Catania, Italy; 2 Biomedical and Biotechnological Sciences, University of Catania, Via Santa Sofia 97, 95123 Catania, Italy; nunziovicario@unict.it (N.V.); soniacalabrese@hotmail.com (G.C.); parenti@unict.it (R.P.)

**Keywords:** silicon, microarray, biosensors, nucleic acids detection

## Abstract

Surface substrate and chemical functionalization are crucial aspects for the fabrication of the sensitive biosensor based on microarray technology. In this paper, an advanced, silicon-based substrate (A-MA) allowing enhancement of optical signal for microarray application is described. The substrate consists in a multilayer of Si/Al/SiO_2_ layers. The optical signal enhancement is reached by a combination of the mirror effect of Al film and the SiO_2_ thickness around 830 nm, which is able to reach the maximum of interference for the emission wavelength of the Cy5 fluorescent label. Moreover, SiO_2_ layer is suitable for the immobilization of single-strand DNA through standard silane chemistry, and probe densities of about 2000 F/µm^2^ are reached. The microarray is investigated in the detection of HBV (Hepatitis B Virus) pathogen with analytical samples, resulting in a dynamic linear range of 0.05–0.5 nM, a sensitivity of about 18000 a.u. nM^−1^, and a Limit of Detection in the range of 0.031–0.043 Nm as a function of the capture probe sequence.

## 1. Introduction

Microarrays (MAs) are one of the most powerful and widely used biotechnologies for nucleic acids detection and are particularly relevant in the field of genomics [1]. Currently, MAs are attracting great interest among biologists, biochemists, and biomedical researchers, mainly for gene expression evaluation and mutation detection [2,3]. Besides detecting nucleic acids, MAs are also widely used to analyze proteins [4,5], carbohydrates [6], and cells [7]. The strengths of this technology are high throughput, miniaturization, and multiplexing analysis [8,9]. The integration of microarray technology with microfluidics, microelectronics, MEMS, and optical technologies has led to the development of modern and advanced miniaturized Lab-on-chip (LoC) devices [10,11,12]. In this context, silicon material offers several advantages for creating technological advancements in smart LoC devices. Actually, it combines significant physical properties (such us good electrical, thermal, and photoconductivity) with important technological aspects including consolidated production technologies, industrialization processes, and the integration of “intelligence on board” through microelectronic circuitry [13,14,15]. 

MA consists of a large amount of arrayed capture probes anchored by a proper chemistry to the surface of a solid substrate. In case of DNA detection, the capture probes are single-strand oligonucleotides, which are generally 25 mer in length (ssDNA microarray). The fabrication processes can be summarised in two steps: (a) *in situ* synthesis of the capture probe and (b) the microspotting of pre-synthesized capture probes by contact or noncontact printing technologies [16]. In this context, the probe immobilization strategy plays a key role, strongly influencing probe density and, then, hybridization processes [17,18,19]. Many chemistry strategies suitable for covalent capture probe attachment are reported in the literature, such as epoxy-amine, aldehyde-amine, metal-sulphur, biotin-streptavidin, and amine-*N*-hydroxy succinimide chemistries [20,21]. Similarly, unmodified oligonucleotides can bind to positively charged amine-functionalized substrates, such as chitosan, poly-lysine, or others, to obtain an electrostatically surface attachment [20]. Once prepared, ssDNA microarrays will be hybridized through a reaction in which the capture probes molecularly recognize the complementary sequence of an amplified DNA target (polymerase chain reaction, PCR product) or, recently, of a whole genome without PCR amplification [22]. 

The hybridization response can be transduced by various methods such as magnetic [23], optical [24], mechanical [25], or electrochemical signals [26]. Due to the relative high sensitivity, the most common transduction method currently in use is the fluorescence quantification of molecular dyes, such as Cy5, Cy3, VIC, and FAM, which label the DNA target upon the hybridization process. An interesting approach to increase the optical signal for the detection of biomolecules was proposed by Zhao [27]. The method is mainly based on porous silicon microcavity that is able to change the effective refractive index upon the molecular recognition event and to increase the surface area for the molecular capture.

Although the analytical performances of ssDNA microarrays such us Limit of Detection (LoD), dynamin range, and sensitivity are correlated with (i) the specific capture probe (sequence and length), (ii) probe density (strategy of immobilization), and (iii) the experimental hybridization protocols, they are also strongly affected by the level of fluorescence emission upon the probe-to-target recognition process.

In this work, an ssDNA MA based on an advanced silicon-based substrate A-MA that is able to enhance the hybridization fluorescent signal for the detection of Hepatitis B Virus (HBV) specific sequence is reported. The substrate consists of a multilayer of Si/Al/SiO_2_ layers. The Al film acts as an optical mirror, while the SiO_2_ layer is properly modulated in its thickness to achieve fluorescence enhancement by constructive interference of Cy5 emission. The evaluation of signal enhancement, together with the system analytical perfomances in terms of dynamic linear range, sensitivity, LoD, and LoB (Limit of Background), are presented and discussed.

## 2. Materials and Methods

### 2.1. Chemicals

All the reagents used for the chemical process, microarray fabrication, and testing experiments were purchased by Sigma-Aldrich and used as received. Bovine serum albumin (BSA) fraction V was purchased from Euroclone.

The oligonucleotide 5′amino-derivatised ssDNA probes P1 (5′H_2_N-C6-GGT GAG TGA TTG GAG GTT), P2 probe: (5′H_2_N-C6-CAC ATC AGG ATT CCT AGG), P1-Cy5 labelled probe: (5′H_2_N-C6-GGT GAG TGA TTG GAG GTT-3′Cy5) and negative control (Pneg) probe (5′H_2_N-C6-AAA AAA AAA AAA AAA AAA) were purchased from MWG (Germany). The complementary hybridization Cy5 labeled target P1* (Cy5 5′-AAC CTC CAA TCA CTC ACC) and hybridization Cy5 labeled target P2* (Cy5 5′-CCT AGG AAT CCT GAT GTG) were purchased by MWG Biotech.

### 2.2. Microarray Fabrication

The A-MA microarray substrate (7.5 × 2.5 cm) was manufactured by standard VLSI technology and consists of a silicon substrate (about 600 μm of thickness) featured by a multilayer of Al film placed by CVD method (900 nm) and a SiO_2_ layer placed by TEOS precursor with PE-CVD technique, with thickness ranging from 780 nm to 925 nm.

**Figure 1:**
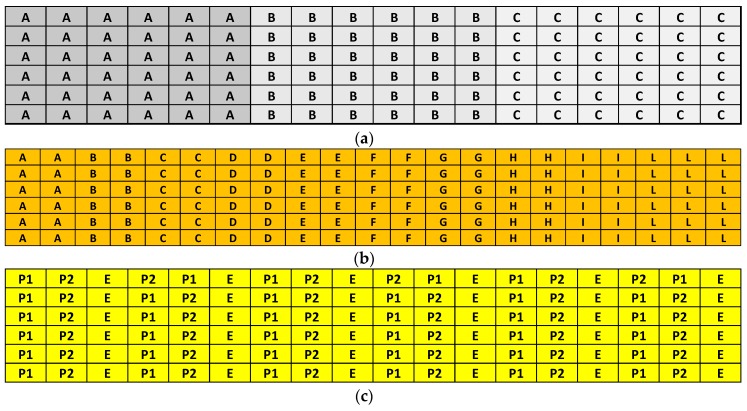
(**a**) ssDNA microarray layout for optical characterization containing different concentrations of Cy5-labelled oligo P1 A = 0.1 µM; B = 0.01 µM; and C = 0.001 µM; (**b**) ssDNA microarray layout for probe density characterization containing different concentration of Cy5-labelled oligo P1 A = 5000 F/µm^2^, B = 2000 F/µm^2^, C = 1000 F/µm^2^, D = 800 F/µm^2^, E = 500 F/µm^2^, F = 300 F/µm^2^, G = 100 F/µm^2^, H = 50 F/µm^2^, I = 25 F/µm^2^, and L = 10 F/µm^2^; (**c**) ssDNA microarray layout for analytical performances: P1 = P1 probe; P2 = P2 probe; E = empty position.

(a) *Chemical process*. The A-MA substrates were chemically processed with the following steps: *(a)* cleaning (to remove organic contamination) and activation process (to increase the Hydroxyl group (-OH) density at SiO_2_ surface) by plasma O_2_ treatment was performed by Sentech plasma generator (300 s, 100 W); *(b)* vapor phase silanization with GOPS (glycidoxysilane) was carried out in under vacuum Oven with the follow conditions: 4 h, 0.1 Atm, and 125 °C; *(c)* spotting of capture probes by a Perkin Elmer piezo spotter dispensing drops of 300 pL of solution of oligonucleotides (10 µM) in Na_2_HPO_4_ buffer (150 mM, pH = 9); *(d)* probe anchoring step by incubating the spotted substrate for 4 h at 30 °C and 90% humidity relative in a climatic cell; *(e)* passivation step by deposition of BSA protein layer, dipping the substrates in BSA 1%aqueous solution, SSC 2X, SDS 0.1%, for 15 h at 55 °C. The substrates were then rinsed in deionized water and dried by a nitrogen flow. Each process step was characterized by XPS analysis (see below Section 3.2.1).
(b) *Microarray for optical characterization*. 6 × 14 microarray layout (spot size 100 µm, pitch 250 µm) was prepared by a Non-Contact PiezoArray (PERKIN ELMER) in a clean room. The spotting plate for this study was composed of two main DNA oligo solutions made by a mixture of Cy5-labeled oligo P1 and not-labeled oligo P1 at different concentration ratios to maintain the total concentration of DNA oligo ([Cy5-labeled oligo P1] + [not-labeled oligo P1] ) equal to 10 µM in 150 mM of phosphate printing buffer. Figure 1a reports the scheme of the layout used for this study.
(c) *Microarray for probe density characterization*. Microarray layout of size 6 × 21 (spot size 100 µm, pitch 250 µm) was prepared by a Non-Contact PiezoArray (PERKIN ELMER) in clean room. The spotting solutions were composed of two main DNA oligo solutions made by a mixture of Cy5-labeled oligo P1 and not-labeled oligo P1 at different concentrations to maintain the total concentration of DNA oligo ([Cy5-labeled oligo P1] + [not-labeled oligo P1] ) equal to 10 µM in 150 mM of phosphate printing buffer. The final concentrations of P1 Cy5-labeled probes were in the range of 5000–10 F/µm^2^. Figure 1b reports the scheme of the layout used for this study.
(d) *Microarray for analytical performances characterization*. Microarray layouts of size 6 × 18 spots (spot size 100 µm, pitch 250 µm) were printed by a Non-Contact PiezoArray (PERKIN ELMER) in clean room according to previously described procedures [28].

### 2.3. Hybridization Protocol

The hybridization reaction was carried out by a 250 μL solution containing P1* Cy5 and P2* Cy5 labelled perfect match targets at various concentrations of 0.0005 nM, 0.005 nM, 0.05 nM, 0.10 nM, 0.50 nM, 5.00 nM, 15.00 nM, and 50.00 nM in 20 mM of sodium phosphate buffer, 1 M NaCl, 5.2 mM fo KCl, 0.1% of Tween20, 2 × of Denardt’s solution, and 20 μg/μL of ssDNA. After hybridization, a washing step consisting of 5′ at 40 °C in 2X SSC, followed by a second wash of 5′ at 40 °C in 0.2X SSC, was carried out.

### 2.4. SiO_2_ Thickness and Optical Measurements

The measurements of SiO_2_ thickness and the reflectance spectra were performed by Ellipsometer SENTCH850.

The microarray fluorescent images were acquired by Optical Reader SN 038 [3], set in multisot 5 acquisition mode. Images were analyzed by a customized software InCheck Platform H-MAT version 2.5.1 (developed by STMicroelectronics) with the following analysis protocol. For each spotted slide, the average and standard deviation of signal intensity (σ) of the 42 replicas of each Cy5-labled oligo DNA concentration of layout in Figure 1 was calculated. These values were correlated with the specific SiO_2_ thickness.

## 3. Results and Discussion

### 3.1. Substrate Optical Characterization

**Figure 2:**
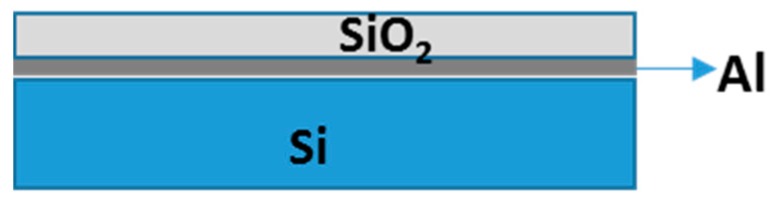
A-MA Substrate Multilayer structure for fluorescence enhancment.

The A-MA substrate structures were composed of a multilayer structure comprising Silicon substrate, on top of which was placed a layer of Al and SiO_2_ film with thickness of about 830 nm (Figure 2). This structure has been designed to allow enhancement of light occurring at the substrate surface, since SiO_2_ film thickness was modulated as function of Cy5-emitted light, and Al acts as mirror.

#### 3.1.1. Reflectance Measurements

**Figure 3:**
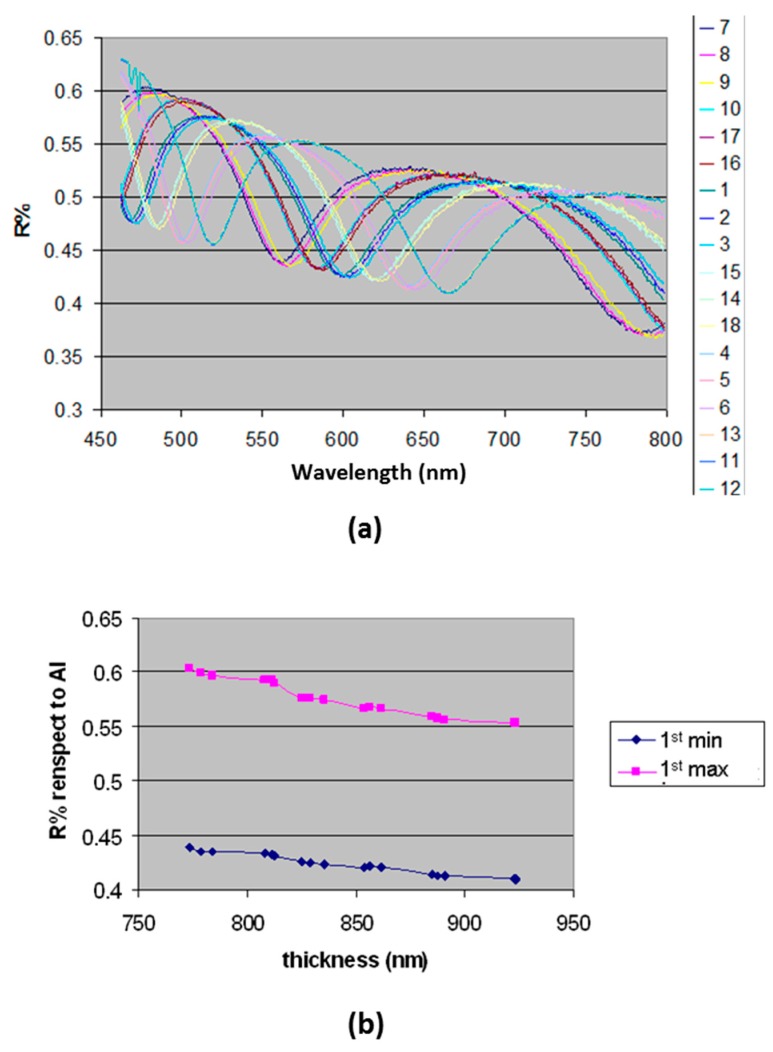
(**a**) Reflectance Spectra substrates with SiO_2_ thicknesses ranging between 770 nm and 925 nm; (**b**) R% values at the first interference maximum and minimum.

In order to evaluate the mirror effect of the Al layer in the multilayer structure of the A-MA substrate, reflectance measurements were carried out on substrates featured by SiO_2_ layers with thicknesses ranging between 780 nm and 925 nm. The obtained reflectance spectra were expressed by percentage of reflectance variation (R%) compared to the signal of the reference sample (i.e., pure Al layer) (Figure 2a). The R% values at the first interference maximum and minimum are plotted in Figure 3b. 

The R% variation over the explored wavelength range in the case of both 1st max (from 0.6 to 0.55) and 1st min (from 0.44 to 0.41) returns in the range of variability of the signal of Al reference sample within the explored wavelength range.

This finding indicates that the variation of the Al metal reflectivity is negligible over the explored SiO_2_ thickness range with a consequent negligible variation of the fluorescence signal.

#### 3.1.2. Fluorescence Signal Characterization

**Figure 4:**
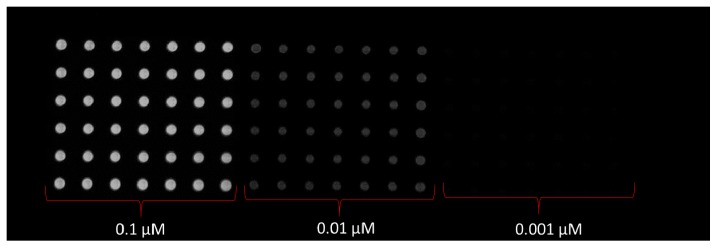
Microarray layout containing different concentration of P1-Cy5 labeled probes: 0.1 µM; 0.01 µM and 0.001 µM.

In order to characterize the level of light enhancement on Cy5 fluorescence signals, three concentrations (60 replica each conctentration) of P1-Cy5 labeled probes 0.1 µM (20 fluorophores µm^−2^), 0.01 µM (200 fluorophores µm^−2^), and 0.001 µM (2000 fluorophores µm^−2^ ) were spotted onto A-MA surfaces of substrates with SiO_2_ thicknesses ranging between 780 nm and 925 nm. Figure 4 illustrates the obtained microarray fluorescence image, highlighting also the relative capture probes layout.

**Figure 5:**
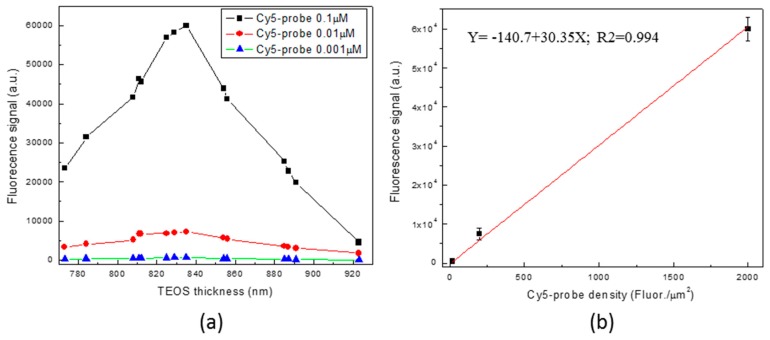
(**a**) P1 Cy5-probe fluorescence intensities versus SiO_2_ thickness and (**b**) linear correlation between the Cy5-probe density (fluorophores/µm^2^) versus the fluorescence signal intensity (Y = −140.7 + 30.35X; R^2^ = 0.994).

The values of fluorescence signal intensities versus the SiO_2_ thickness for all the three concentrations of P1 Cy5-probe investigated (0.1 µM, 0.01 µM, and 0.001 µM) are reported in Figure 5. The curve shows a maximum of interference of about 835 nm with a width of 90 nm at half maximum. Data of concentration 0.1 µM were fitted by the mathematical model based on the Sinesqr Equation (1):

Y = A sin^2^(π (x − xc)/w) (1)

in which A is the difference of signal between max and min, xc (725 ± 6 nm) is the x position of min, w is the full width at half maximum (210 ± 10 nm), Y is the signal recorded (52650 ± 1700 a.u.) and x is the SiO_2_ thickness (830 ± 8 nm). The fitting shows a maximun of fluorescence peak at 830 ± 8 nm and a bandwidth value of 105 ± 20 nm. These data confirm the experimental finding above reported showing a maximum of interference for the Cy5 emission wavelength at a SiO_2_ thickness value of about 830 nm with a width of 90 nm at half maximum.

The linear correlation between the Cy5-probe densities (20, 200, and 2000 fluorophores/µm^2^ calculated assuming spot size of about 110 µm^2^) versus the fluorescence signal intensities measured at the maximum of interference of 835 nm is reported in Figure 4b. Data show a significant linear fit (Y = −140.7 + 30.35X; R^2^ = 0.994). The limit of detection (LoD) has been calculated using as Y value a current value corresponding to four-times the background signal (116 a.u.). A LoD of about of about 20 fluorophores/µm^2^ was found.

### 3.2. Surface Grafting Characterization

#### 3.2.1. Chemical Process and Grafting Characterization

**Figure 6:**
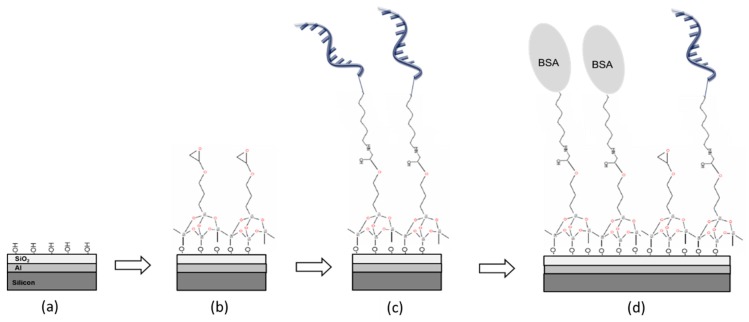
Scheme of chemical process: (**a**) plasma-O_2_ cleaning and activation, (**b**) epoxy-silanization, (**c**) capture probe spotting and anchoring, and (**d**) BSA passivation process.

Figure 6 reports the scheme of the chemical steps used in the present study for the grafting of the ssDNA probes that comprises (a) plasma-O_2_ cleaning and activation, (b) epoxy-silanization, (c) capture probe spotting and anchoring, and (d) BSA passivation. In order to follow the effectiveness of the chemical process, each step was characterized by XPS analysis. After plasma-O_2_ process (step (a)), the XPS spectra shows the diagnostic peaks for Si2p (30%) and O1s (56.5%), proving the goodness of the cleaning. Upon silanization (step (b)), the XPS spectrum in the range of 0–1000 eV exhibits the specific peaks for Si2p (27%), O2p (51.6%), and C1s (10%). A Gaussian-Lorentz deconvolution for the C1s peak reports two main components: a first centered at about 285.2 eV attributed to C-C and C-H carbon species and a second centered at about 286.7 eV attribute to C-O species. The ratio C1s/Si2p value for silanized surface is 0.37. This is diagnostic of the presence of organic layer covering the substrate surface in line with epoxy-silane derivatization. Finally, after passivation with BSA protein (step (d)), the XPS spectra show the following peaks: C1s at 285 eV (17.31%), O1s (53.70), N1s at 400 eV (2.46%), and Si2p (24.77%). Both the peak for nitrogen (N1s) and the ratio C1s/Si2p ratio of about 0.7 (versus C1s/Si2p ratio of 0.37 for the silanized surface) confirm the presence of a protein layer.

#### 3.2.2. Capture Probe Density Evaluation

**Figure 7:**
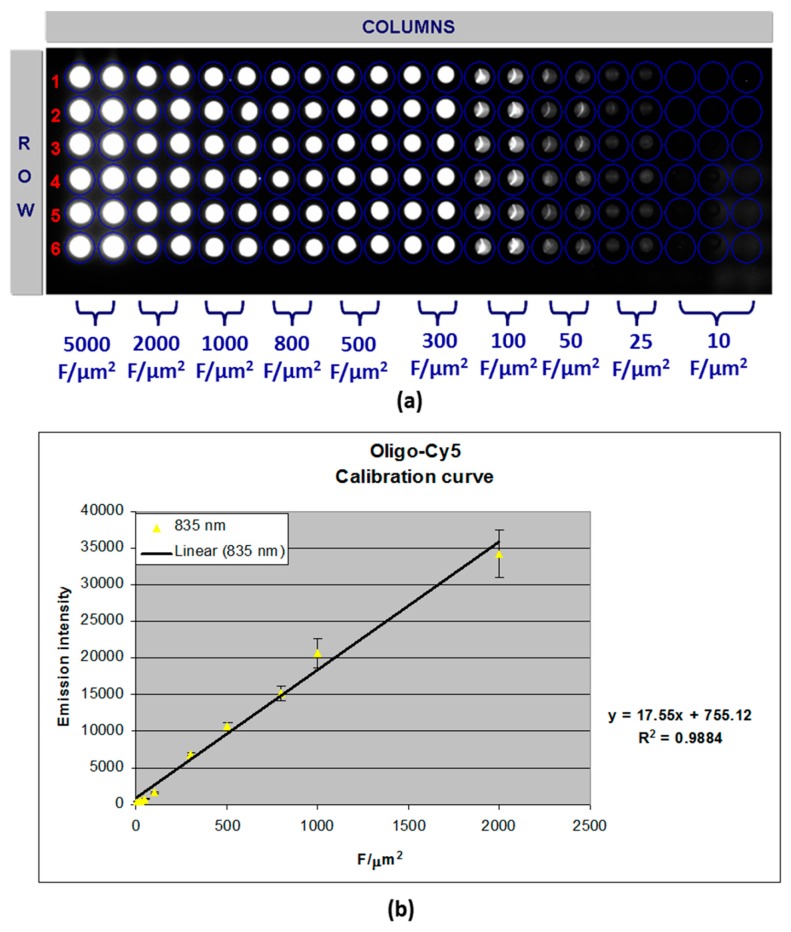
(**a**) Fluorescence image of a panel containing 10 concentrations (5000–10 F/µm^2^) of P1 Cy5 DNA-probe (image acquired by Optical Reader (2000 ms)) and (**b**) calibration curve of signal fluorescence intensities versus Cy5-labeled oligoprobes concentration (F/µm^2^).

In order to evaluate the A-MA surface grating ability, intra-spot probe densities average values were assessed by means of a calibration curve. This curve was obtained by spotting onto A-MA substrate 10 different concentrations (5000–10 F/µm^2^) of P1 Cy5-labeled probes and by recording fluorescence images (Figure 7a). Signal fluorescence intensities extracted by the images were plotted versus Cy5-labeled oligoprobes concentration (F/µm^2^) to obtain the calibration curve illustrated in Figure 7b. A good liner correlation was found (Y = 755.12 + 17.55X; R^2^ = 0.9884). The curve was then used to evaluate the probe density values as described below.

To evaluate the probe density values, A-MAs were prepared by spotting Cy5-labeled P1 and P2 probes according to the chemical process described in the previous section and the layout reported in Figure 1b. The obtained fluorescence images were analysed, and the related signals used to read the corresponding probe density values (F/µm^2^) using the calibration curve above described. Table 1 reports the obtained results for both probes Cy5-labeled P1 and P2. Values around 2000 fluorophores per µm^2^ were found. This finding confirms previously published studies on probe grafting on silicon oxide surfaces [17,19].

### 3.3. Analytical Performances Characterization

The A-MA analitycal performance in terms of linearity, sensitivity LoD, and LoB was assessed performing hybridation with P1* and P1* Cy5 labelled perfect match targets at various concentrations of 0.00 nM, 0.0005 nM, 0.005 nM, 0.05 nM, 0.10 nM, 0.50 nM, 5.00 nM, 15.00 nM, and 50.00 nM, respectively. The Foreground Median (F_Med_) as the median of pixel intensity of fluorescence signal and the Background Mean (B_M_) as the mean of pixel intensity of local background were measured from each hybridized spots. 

**Figure 8:**
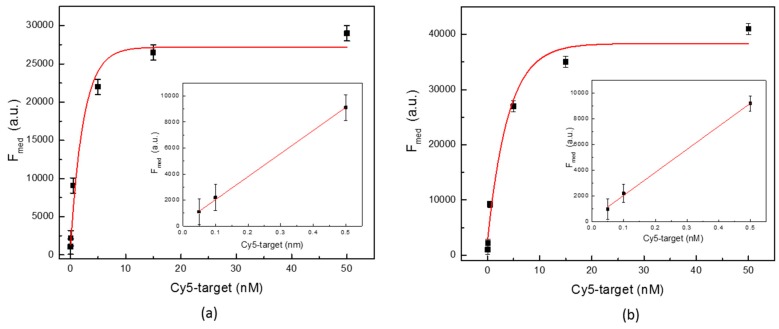
(**a**) Foreground Median Hybridization signal for P1 at various Cy5-target concentration. Inset: the linearity range (Y = 267 + 17702.2X; R^2^ = 0.9985) and (**b**) Foreground Median Hybridization signal for P2 at various Cy5-target concentration. Inset: the linearity range (Y = 270 + 17882.7X; R^2^ = 0.9981).

Figure 8 illustates the average F_Med_ values obtained for both P1 and P2 perfect match over the explored concentration range. Good linearity can be observed for the range between 0.05 nM and 0.5 nM for both specific targets.

In order to calculate both the LoD and the LoB for each probes, a methodology based on linear regression has been applied. More in details, the LoB value for each probe has been found by appliying Equation (2), in which NB is number of background replica and estimating the 95% percentile p = (100 − α) = 95.

LoB = [NB(p/100) + 0.5](2)

From this procedure was obtained for P1 and P2 probes LoB values of about 204 and 257, respectively.

The LoD was estimated set to 4 times the LoB signal intensity value (Cut-off) in the regression linear equation above reported. The finding LoD are 0.031 nM and 0.043 nM for specifc P1 and P2, respectively. Regarding the sensitivity, it was defined as the slope of the regression linear equation, and a value was found of 17702.2 a.u. nM^−1^ for the specific P1 probe and 17882.7 a.u. nM^−1^ for P2, respectively.

## 4. Conclusions

In this work, an advanced, silicon-based substrate (A-MA) able to sensitively enhance fluorescent signals for microarray applications has been presented. The substrate contains a multilayer structure consisting of an Si substrate with Al film acting as a light mirror, and an SiO_2_ layer properly modulates the thickness (835 nm) to achieve the fluorescence enhancement by light constructive interference for the emission wavelength of the Cy5 fluorescent label. The immobilization of the ssDNA probe was achieved by means of standard silane chemistry, and probe densities of about 2000 F/µm^2^ were found. The A-MA investigated in the detection of HBV (Hepatitis B Virus) pathogen showed a dynamic linear range of 0.05–0.5 nM, a sensitivity of about 18000 a.u. nM^−1^, and a Limit of Detection in the range of 0.031–0.043 nM as a function of the capture probe sequence.

In conclusion, the main strength of the A-MA substrate can be summarized as it follows: (i) the total reflection of fluorescence-signal; (ii) the good optical transmission of SiO_2_ layer; (iii) the ability to modulate the SiO_2_ thickness to gain the maximum interference in correlation to the fluorescence-label emission wavelength; and (iv) the high chemically versatility of SiO_2_ surface, being easily modified by silane-strategy to obtain covalent capture probes anchoring. All these features make this substrate very promising in the development of new microarray platforms for the next generation of molecular diagnostic devices.

## Figures and Tables

**Table 1:** Probe Density Values (F/µm^2^) for Cy5-labeled P1 and P2 probes.

Probe.	Fluorescence (a.u.)	Probe Densities (F/µm^2^)
Cy5-P1	32300 ± 1500	1815 ± 120
Cy5-P1	34620 ± 2100	2020 ± 150
